# Compound-specific recording of gadolinium pollution in coastal waters by great scallops

**DOI:** 10.1038/s41598-019-44539-y

**Published:** 2019-05-29

**Authors:** Samuel Le Goff, Jean-Alix Barrat, Laurent Chauvaud, Yves-Marie Paulet, Bleuenn Gueguen, Douraied Ben Salem

**Affiliations:** 1 Laboratoire Géosciences Océan (UMR CNRS 6538), Université de Bretagne Occidentale et Institut Universitaire Européen de la Mer (IUEM), Place Nicolas Copernic, 29280 Plouzané, France; 2 Laboratoire des Sciences de l’Environnement Marin (UMR CNRS 6539), LIA BeBEST, Université de Bretagne Occidentale et Institut Universitaire Européen de la Mer, Place Nicolas Copernic, 29280 Plouzané, France; 3 UMS CNRS 3113, Université de Bretagne Occidentale et Institut Universitaire Européen de la Mer, Place Nicolas Copernic, 29280 Plouzané, France; 4 LaTIM (INSERM UMR 1101) Université de Bretagne Occidentale. 22, avenue C. Desmoulins, 29238 Brest Cedex 3, France

**Keywords:** Environmental impact, Environmental impact, Element cycles, Element cycles

## Abstract

Gadolinium-based contrast agents (GBCAs), routinely used in magnetic resonance imaging (MRI), end up directly in coastal seawaters where gadolinium concentrations are now increasing. Because many aquatic species could be sensitive to this new pollution, we have evaluated the possibility of using shellfish to assess its importance. Gadolinium excesses recorded by scallop shells collected in Bay of Brest (Brittany, France) for more than 30 years do not reflect the overall consumption in GBCAs, but are largely controlled by one of them, the gadopentetate dimeglumine. Although its use has been greatly reduced in Europe over the last ten years, gadolinium excesses are still measured in shells. Thus, some gadolinium derived from other GBCAs is bioavailable and could have an impact on marine wildlife.

## Introduction

For thirty years now, linear or macrocyclic gadolinium-based contrast agents (GBCAs) are routinely used in magnetic resonance imaging (MRI)^1^. They were initially assumed to have virtually no side effects, but gadolinium can accumulate in tissue, bone and brain^2–4^. Moreover, linear GBCAs cause nephrogenic systemic fibrosis, a debilitating and potentially life-threatening disease, for patients with kidney failure^5,6^. Thus, the use of some linear GBCAs has been reduced over the last decade, and recently banned in Europe^7^. As GBCAs are not recovered during wastewater treatment, more than 200 tons of anthropogenic gadolinium are conveyed each year by rivers of developed and densely populated areas, and end up directly in coastal seawaters where gadolinium concentrations, still low, are now increasing^8–12^. Aquatic species are sensitive to this new pollution: for example, gadolinium accumulates in the livers of fish and induces antioxidant enzyme productions^13^; in presence of gadolinium, larvae of sea urchins display abnormal shapes and growth^14^; gadolinium exposure produces mitochondrial and anti-inflammatory pathways in freshwater mussels^15^.

**Figure 1:**
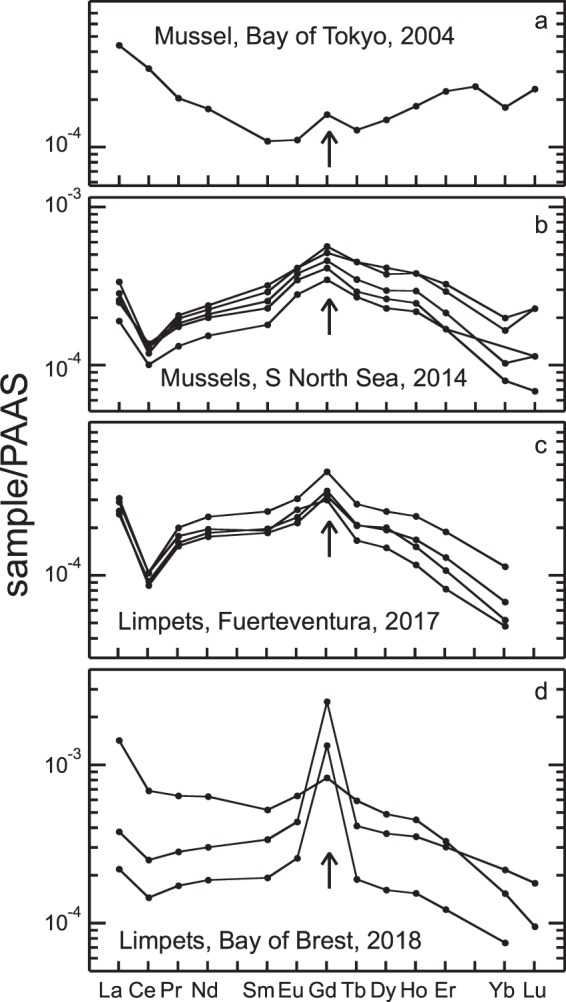
REE patterns of shells normalised to Post Archean Australian Shale (PAAS)^19^, from Japan^16^ (**a**), North Sea^17^ (**b**), Fuerteventura, Canary Islands (**c**), and Bay of Brest, France (**d**).

The incorporation of anthropogenic gadolinium by shellfish is evidenced by their Rare Earth Elements (REE) patterns. Unlike cerium which has two valence states in aqueous systems, and is easily decoupled from lanthanum and praseodymium, its neighbouring REEs, gadolinium has only one trivalent valence state, and cannot be easily decoupled from other rare earth elements by natural processes. Thus rocks and unpolluted waters do not show any large anomalies in gadolinium. Some mollusc shells from densely populated coastal areas such as Tokyo Bay^16^ or the southern North Sea^17^ now display positive gadolinium anomalies explained by pollution. Similar anomalies are also measured in shells from the Canary Islands and the French coasts, showing that this pollution affects vast regions (Fig. 1). Consequently, coastal shellfish could represent valuable archives of this pollution. Among non-motile shellfish, the great scallop (*Pecten maximus*) could be considered as a fixed sensor recording environmental variations because of its longevity (up to 12 years), high growth rate of its shells (up to 350–400 µm/day during growing phase) and production of both seasonal and daily growth bands^18^. We have studied a collection of wild great scallops collected alive during the last thirty years at the same sampling site of Bay of Brest at a mean depth of 20 m (Supplementary Fig. 1). All these scallops were fished in late fall and were between 3 and 4 years old (estimated with winter marks, see Fig. S2). Their sizes ranged from 8 to 10 cm. We analysed fragments of their left valves, corresponding to the carbonate formed since the winter preceding their catch.

## Results and Discussion

**Figure 2:**
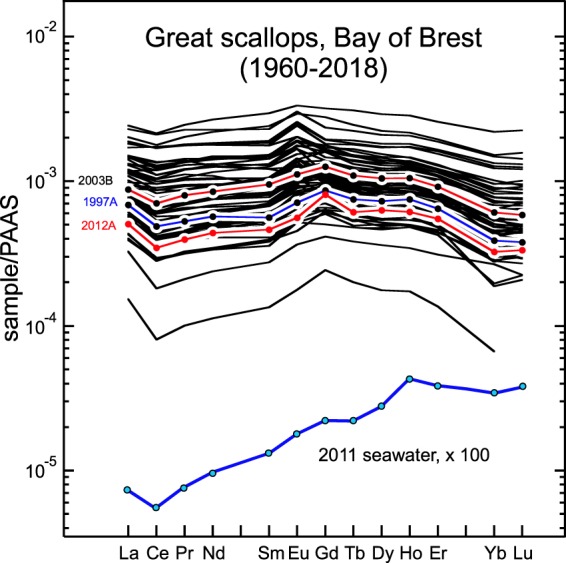
REE patterns of scallop shells from Bay of Brest normalised to Post Archean Australian Shale (PAAS)^19^. The pattern of the local seawater is shown for comparison^21^.

**Figure 3:**
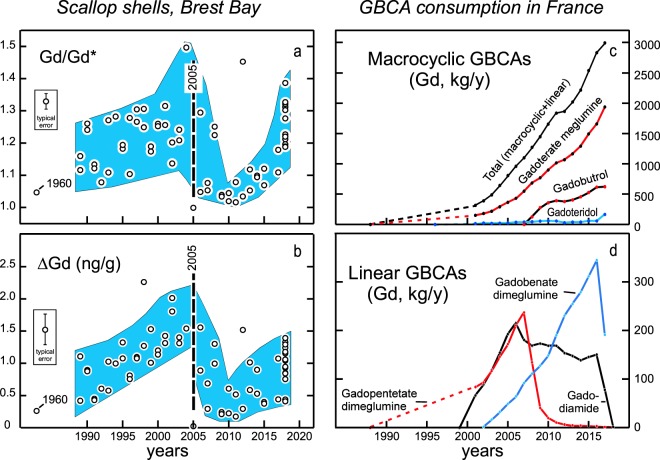
Gadolinium anomalies (**a**: Gd/Gd*), gadolinium excesses (**b**: ΔGd) recorded by scallop shells sampled from 1960 to 2018 in Bay of Brest, and GBCA consumption in France recorded in the Medic’AM database^22^ maintained by the CPAM (French Health Insurance agency) (**c**: total consumption and macrocyclic GBCAs, **d**: linear GBCAs).

The abundances of REEs in shells Fig. 2 and Supplementary Table 2) are low and highly variable: they range between 6 × 10^−5^ and 3 × 10^−3^ times the shale reference^19^. The shapes of the REE patterns are similar to those of other coastal shellfish (e.g., Fig. 1), with variable negative cerium anomalies (Ce/Ce* = 0.65–1.33, see Methods for the calculations of REE anomalies). In bivalves, shells result from the activity of the mantle epithelium inside specific internal liquid^20^. Their components are derived from organic matter, inorganic particles and water that have been assimilated by the mollusks^16^. Thus, their REE patterns are different from those of local seawater^21^, with less marked LREE depletions relative to shales. Furthermore, they display small but significant positive gadolinium anomalies (Gd/Gd* = 1.00–1.50, Fig. 3), which indicate that shells incorporated small amounts of anthropogenic gadolinium.

The gadolinium anomalies (Gd/Gd*) in scallop shells do not show a clear temporal evolution (Fig. 3). Since shells have very different levels of REE concentrations, a given amount of anthropogenic gadolinium can result in very different Gd/Gd* ratios in carbonates. To reduce this effect, we estimated the gadolinium excess in each sample using the difference between the measured gadolinium concentration and the interpolated one (ΔGd = Gd − Gd*). These excesses in scallop shells (ΔGd = 0–2.3 ng/g) display a complex temporal evolution (Fig. 3b). The oldest sample collected in 1960, before the use of GBCAs, does not show any significant excess in gadolinium. A marked increase in gadolinium excesses is seen from 1989 to 2005, followed by a sharp decline until 2010 when normal levels are observed again. Afterwards, the excesses seem to increase again without reaching the 2005 maximum, but the data show some spread. Such an evolution is unexpected because the use of GBCAs has always been increasing since their introduction on the market. It could depend on the bioavailability of anthropogenic gadolinium as determined by its speciation in seawater.

Over the last thirty years, various GBCAs have been used. Their behaviours in seawater could be very different. The quantities of GBCAs produced by the different pharmaceutical companies and distributed each year in the different regions of the world are not in the public domain. However, these figures can be deduced for the French market: GBCAs can only be issued upon the prescription of a health care professional, dispensed by a licensed pharmacist, and are reimbursed by the Caisse Primaire d’Assurance Maladie (CPAM), the French health insurance agency. We used the CPAM database^22^ to assess the number of reimbursed boxes of referenced GBCAs each year since 2001. These data are not available for years before 2001, and we interpolated previous consumption using marketing authorisation dates. Although some prescriptions may have escaped this database, it provides a reliable picture of annual consumption of GBCAs and the relative importance of the different molecules (Fig. 3c,d). Prior to 2007, three GBCAs were mainly used in France: one macrocyclic, the gadoterate meglumine, and two linear GBCAs, the gadopentetate dimeglumine and the gadodiamide. When the link between nephrogenic systemic fibrosis and the administration of linear GBCAs was suspected around 2005^5,6^, the prescriptions of some of them were considerably reduced, notably the gadopentetate dimeglumine. The sudden reduction of the gadolinium excesses in the shells is consistent with the abandonment of this agent. However, an apparent discrepancy between the decrease in French consumption of this agent (in 2007) and that of the Gd excesses recorded by the shells (in 2005) should be noted: prescribers in Western Brittany could have chosen another contrast agent as soon as 2005, either in accordance with a precautionary principle or due to supply or cost constraints. The available archives do not allow for a more detailed discussion of this point which should therefore not be overemphasized. Today, gadolinium consumption by MRI in France is three times that of 2005, but the magnitude of this increase is not recorded by scallop shells (Fig. 3). This could suggest that the bioavailability of anthropogenic gadolinium currently present in coastal areas is low.

GBCAs are very stable in fresh water. Anthropogenic gadolinium seems not to be very reactive and can be used as a useful tracer of the effluents of water-waste treatment plants^23^. Its bioavailability is apparently low. Accumulations of gadolinium have been detected in digestive glands and gills of a few fresh water bivalves^24^, but their shells do not show noticeable positive gadolinium anomalies^25^, suggesting that the GBCA molecules have been degraded little or not at all by these molluscs. The situation is different in the sea, but few data exist on the stability of GBCAs in marine waters. At present, only gadopentetate dimeglumine has been studied in depth^26^. This contrast agent is substantially dissociated upon mixing of river water with seawater in estuaries. Once these molecules are degraded, the gadolinium associated with them can become bioavailable and possibly toxic. This explains the incorporation of anthropogenic gadolinium by scallop shells seen until 2005–2006. The lack of a clear correlation between the gadolinium excesses recorded by the shells and the consumption of the other GBCAs from 2010 to today (Fig. 3) could suggest that the latter, particularly the macrocyclic GBCAs that are currently the most used, are extremely stable in seawater. However, although the use of linear GBCAs is now marginal in France (10% of gadolinium used in MRI was in the form of linear GBCAs in 2017), gadolinium excesses are still measured in shellfish (Figs 1 and 3). Two hypotheses can explain this observation: 1/part of the gadolinium derived from the degradation of linear GBCAs was adsorbed by sediments (clays, iron oxyhydroxides…) before 2005, and is now released; 2/ most of the bioavailable anthropogenic gadolinium still derives today from the destabilization of linear GBCAs, namely gadobenate dimeglumine which was seldom used before 2010, or from macrocyclic GBCAs, the degradation of which is probably much slower in the marine environment than that of gadopentetate dimeglumine. The first hypothesis is unlikely because anthropogenic gadolinium is apparently not affected by any kind of estuarine scavenging^9,10^. Accurate REE analyses on other types of organisms must, of course, be carried out in order to have a more complete view of gadolinium pollution on marine wildlife. However, it is urgent to determine the stability in seawater of the GBCAs currently used, particularly for the most widely used macrocyclic GBCAs such as gadoterate meglumine, gadobutrol and gadoteridol. As the world consumption of these molecules is increasing, these data are essential to predict the consequences of gadolinium pollution in coastal environments and possibly develop water treatment processes.

## Methods

For each sample, about 100 mg of carbonates were spiked with a solution of pure Tm and dissolved in HNO_3_ in a Teflon beaker. REE have been separated from the major elements and concentrated before analysis^27^. Abundances were determined using a high-resolution inductively coupled plasma-mass spectrometer Thermo Element HR at Institut Universitaire Européen de la Mer (IUEM), Plouzané, France. Each sample was analysed in duplicate or in triplicate, and the results were averaged^28^. Results on a carbonate standard obtained during the sessions are given in Supplementary Information.

The Gd anomalies are calculated using the Gd/Gd* ratio, where Gd* is the interpolated Gd concentration for a smooth Post Archean Australian Shale-normalized REE pattern and X_n_ is the concentration of element X normalised to PAAS:

$${{{\rm{Gd}}}^{\ast }}_{{\rm{n}}}={{{\rm{Sm}}}_{{\rm{n}}}}^{1/3}\times {{{\rm{Tb}}}_{{\rm{n}}}}^{2/3}$$

The gadolinium excesses are simply given by the following equation:

$${\rm{\Delta }}\mathrm{Gd}={\rm{Gd}}-{{\rm{Gd}}}^{\ast }$$

Based on standards and sample replicates, the precisions for abundances and element ratios (including Gd/Gd* ratios) are in most cases much better than 5% (2 RSD) (see Supplementary Information). Typical errors for Gadolinium excesses in shells are assumed to be about 0.25 ng/g (2 σ).

## Supplementary information

Supplementary Informations

## Data Availability

All data is available in the main text or the Supplementary Materials.
