# Supplementary material for: Compound-specific recording of gadolinium pollution in coastal waters by great scallops
Source: Sci Rep. 2019 May 29;9:8015. doi: 10.1038/s41598-019-44539-y (PMC6541655; doi:10.1038/s41598-019-44539-y)
Supplement: Supplementary file 1 — Supplementary Informations [file 41598_2019_44539_MOESM1_ESM.docx]

Supplementary Information

for:

**Compound-specific recording of gadolinium pollution in coastal waters by great scallops**

Samuel Le Goff^1^, Jean-Alix Barrat^1*^, Laurent Chauvaud^2^, Yves-Marie Paulet^2^, Bleuenn Gueguen^3^, Douraied Ben Salem^4^

^1^Laboratoire Géosciences Océan (UMR CNRS 6538), Université de Bretagne Occidentale et Institut Universitaire Européen de la Mer, Place Nicolas Copernic, 29280 Plouzané, France.

^2^Laboratoire des Sciences de l’Environnement Marin (UMR CNRS 6539), LIA BeBEST, Université de Bretagne Occidentale et Institut Universitaire Européen de la Mer, Place Nicolas Copernic, 29280 Plouzané, France.

^3^UMS CNRS 3113, Université de Bretagne Occidentale et Institut Universitaire Européen de la Mer, Place Nicolas Copernic, 29280 Plouzané, France.

^4^LaTIM (UMR INSERM 1101) Université de Bretagne Occidentale. 22, avenue C. Desmoulins, 29238 Brest Cedex 3, France

**1/ Sampling areas**

**a/ Great scallops**

Bay of Brest is a semi-enclosed marine ecosystem connected to shelf waters by a 2 km-narrow and 40 m-deep strait (Supplementary figure 1). Two rivers, the Elorn and the Aulne, make up 80% of the total freshwater input in the Bay. All the scallops were fished near Roscanvel at depth > 20 m. In this area, the contribution of freshwater is limited. Bottom water salinity is quite stable from spring to fall (34-35 ‰) and decreases only to 32.5 ‰ during flood tide in winter (S1).


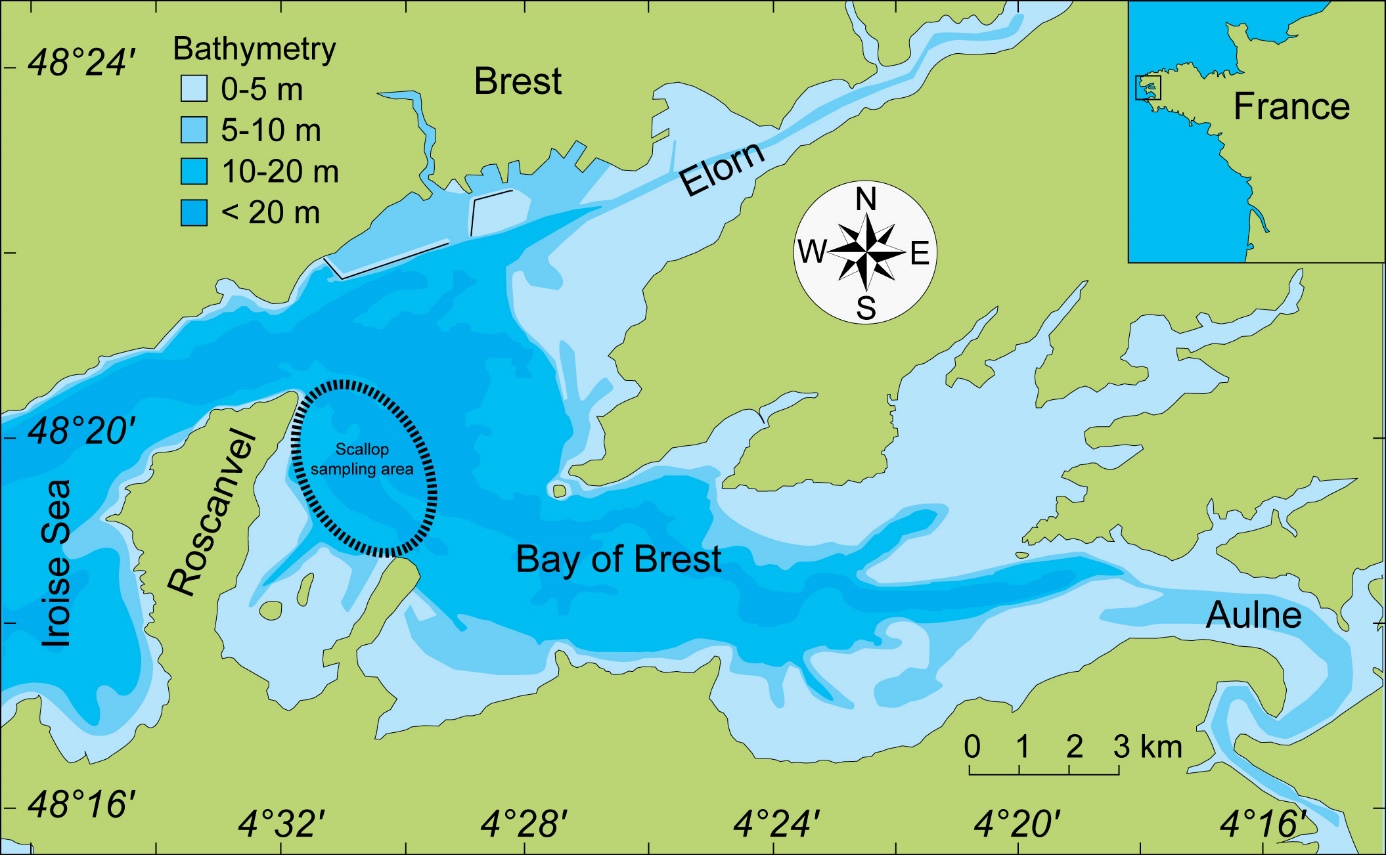


Figure S1. Location of the sampling sites in Bay of Brest (circled area).

b/ Limpets

We analysed limpets from Bay of Brest and from Fuerteventura (Canary Islands). In both cases, shellfish were collected alive.

Bay of Brest: Limpets PMOUL3 and 4 were sampled in February 2018 in the “Moulin Blanc” beach, near Brest. Limpet PM3 was collected the same month on the shore of the island of the dead (“Ile des Morts”), located south of the scallop sampling area.

Fuerteventura: samples were collected in November 2017 in the south of the island.

**2/ Sample preparation**

The left valves of the scallops were abraded with an electric milling cutter to remove any adhering material from the surface, and fragments of their last growth rind were removed (Fig. S2). They were then rinsed with ultrapure water and dried.

Figure S2. Upper surface of the left valve of a great scallop (adult). “Winter marks” (W1 to W4) deposited during spring growth restart are clearly visible, allowing an unambiguous aging.

**3/ Results for the Cal-S standard and shells**

Three tables follow:

Supplementary table 1- results for a carbonate standard.

Supplementary table 2- results for scallop shells.

Supplementary table 3- results for limpet shells.

Supplementary table 1. REE+Y abundances (in ng/g) in the Meuse limestone (prepared by the Service d’Analyses de Roches et des Minéraux (SARM), Nancy) obtained during the course of this study. Each analysis (#1 to 11) was obtained on a separated dissolution.

|  | Y | La | Ce | Pr | Nd | Sm | Eu | Gd | Tb | Dy | Ho | Er | Yb | Lu |  | Gd/Gd* | ΔGd |
| --- | --- | --- | --- | --- | --- | --- | --- | --- | --- | --- | --- | --- | --- | --- | --- | --- | --- |
|  |  |  |  |  |  |  |  |  |  |  |  |  |  |  |  |  |  |
| #1 | 1992 | 764 | 295 | 84.4 | 349 | 61.0 | 15.02 | 88.7 | 13.34 | 95.9 | 25.28 | 79.0 | 64.4 | 9.93 |  | 1.17 | 12.8 |
| #2 | 2005 | 773 | 301 | 85.4 | 353 | 61.8 | 15.16 | 90.0 | 13.56 | 97.4 | 25.67 | 80.0 | 64.6 | 9.89 |  | 1.17 | 12.9 |
| #3 | 2004 | 771 | 291 | 84.5 | 349 | 61.0 | 15.04 | 88.9 | 13.40 | 96.3 | 25.51 | 79.1 | 64.8 | 10.02 |  | 1.17 | 12.8 |
| #4 | 2099 | 815 | 314 | 89.6 | 369 | 64.1 | 15.89 | 92.9 | 13.89 | 99.9 | 26.36 | 81.8 | 65.7 | 9.90 |  | 1.17 | 13.6 |
| #5 | 2087 | 813 | 304 | 88.5 | 365 | 63.1 | 15.70 | 92.9 | 13.80 | 99.0 | 26.17 | 81.3 | 66.0 | 10.00 |  | 1.18 | 14.3 |
| #6 | 2060 | 800 | 294 | 86.9 | 360 | 62.3 | 15.51 | 91.3 | 13.66 | 98.2 | 26.02 | 81.0 | 66.3 | 10.08 |  | 1.18 | 13.6 |
| #7 | 2065 | 790 | 314 | 91.1 | 359 | 63.0 | 15.64 | 91.8 | 13.86 | 99.0 | 26.34 | 82.3 | 67.6 | 10.48 |  | 1.17 | 13.1 |
| #8 | 2120 | 814 | 306 | 88.9 | 367 | 63.7 | 15.84 | 94.0 | 13.98 | 99.6 | 26.38 | 82.1 | 67.4 | 10.30 |  | 1.18 | 14.6 |
| #9 | 2111 | 799 | 308 | 87.2 | 361 | 62.7 | 15.57 | 92.7 | 13.87 | 99.5 | 26.38 | 82.0 | 67.9 | 10.45 |  | 1.18 | 14.0 |
| #10 | 2068 | 786 | 293 | 85.4 | 353 | 61.6 | 15.44 | 91.5 | 13.70 | 98.2 | 25.93 | 80.8 | 67.0 | 10.36 |  | 1.18 | 14.0 |
| #11 | 2101 | 794 | 300 | 86.6 | 359 | 62.5 | 15.61 | 92.3 | 13.82 | 98.7 | 26.17 | 81.5 | 67.2 | 10.35 |  | 1.18 | 13.9 |
|  |  |  |  |  |  |  |  |  |  |  |  |  |  |  |  |  |  |
| average | 2065 | 793 | 302 | 87.1 | 359 | 62.4 | 15.49 | 91.6 | 13.72 | 98.3 | 26.02 | 81.0 | 66.3 | 10.16 |  | 1.17 | 13.6 |
| RSD (%) | 2.2 | 2.2 | 2.6 | 2.5 | 2.0 | 1.7 | 1.9 | 1.9 | 1.5 | 1.4 | 1.5 | 1.4 | 1.9 | 2.3 |  | 0.5 | 4.6 |
|  |  |  |  |  |  |  |  |  |  |  |  |  |  |  |  |  |  |
| Potts (S2) | 1944 | 787 | 333 | 90 | 357 | 64 | 16 | 93 | 14 | 100 | 26 | 81 | 68 | 11 |  | 1.17 | 13.3 |

Supplementary table 2. REE+Y abundances (ng/g) in scallop shells from Bay of Brest. All the analyses were obtained on the last growth rind of different shells.

| # | Y | La | Ce | Pr | Nd | Sm | Eu | Gd | Tb | Dy | Ho | Er | Yb | Lu |  | Gd/Gd* | ΔGd |
| --- | --- | --- | --- | --- | --- | --- | --- | --- | --- | --- | --- | --- | --- | --- | --- | --- | --- |
| 1960-A | 25.25 | 51.71 | 91.31 | 11.30 | 40.68 | 6.35 | 1.28 | 4.89 | 0.63 | 3.32 | 0.60 | 1.48 | 0.96 | 0.13 |  | 1.05 | 0.24 |
| 1989-A | 27.12 | 19.26 | 25.12 | 3.20 | 12.53 | 2.45 | 0.63 | 3.04 | 0.43 | 2.46 | 0.51 | 1.30 | 0.59 | 0.10 |  | 1.16 | 0.42 |
| 1989-B | 61.50 | 65.33 | 108 | 13.80 | 54.09 | 10.47 | 2.30 | 10.64 | 1.43 | 7.83 | 1.54 | 3.99 | 2.87 | 0.44 |  | 1.12 | 1.11 |
| 1990-A | 30.56 | 22.93 | 35.43 | 4.35 | 17.35 | 3.64 | 0.87 | 4.63 | 0.59 | 3.22 | 0.63 | 1.62 | 0.85 | 0.13 |  | 1.24 | 0.90 |
| 1990-B | 28.79 | 23.38 | 35.48 | 4.48 | 16.93 | 3.39 | 0.78 | 4.24 | 0.53 | 2.92 | 0.59 | 1.53 | 0.83 | 0.14 |  | 1.26 | 0.88 |
| 1991-A | 32.99 | 27.20 | 39.55 | 4.95 | 18.77 | 3.21 | 0.74 | 3.82 | 0.54 | 3.15 | 0.65 | 1.71 | 1.02 | 0.14 |  | 1.13 | 0.45 |
| 1991-B | 33.11 | 24.38 | 36.19 | 4.53 | 17.94 | 3.48 | 0.79 | 3.97 | 0.56 | 3.22 | 0.66 | 1.68 | 1.04 | 0.15 |  | 1.12 | 0.43 |
| 1992-A | 53.26 | 58.94 | 105 | 13.86 | 52.82 | 10.11 | 2.03 | 8.39 | 1.07 | 5.80 | 1.15 | 2.99 | 1.83 | 0.25 |  | 1.08 | 0.61 |
| 1993-A | 36.32 | 34.05 | 57.08 | 7.21 | 28.78 | 6.26 | 1.19 | 5.87 | 0.77 | 4.28 | 0.84 | 2.20 | 1.45 | 0.21 |  | 1.11 | 0.58 |
| 1993-B | 29.82 | 25.61 | 38.25 | 4.96 | 19.58 | 3.97 | 0.84 | 4.82 | 0.58 | 3.33 | 0.66 | 1.66 | 1.11 | 0.16 |  | 1.27 | 1.03 |
| 1994-B | 32.11 | 24.04 | 57.85 | 4.60 | 19.57 | 3.56 | 0.71 | 4.54 | 0.55 | 3.23 | 0.64 | 1.66 | 1.00 | 0.14 |  | 1.28 | 1.00 |
| 1995-A | 34.84 | 57.48 | 97.45 | 12.29 | 46.96 | 8.33 | 1.36 | 6.98 | 0.78 | 4.07 | 0.74 | 1.84 | 1.07 | 0.14 |  | 1.18 | 1.08 |
| 1995-B | 45.75 | 40.90 | 66.72 | 8.47 | 34.07 | 7.18 | 1.47 | 8.15 | 1.05 | 5.65 | 1.08 | 2.73 | 1.77 | 0.26 |  | 1.19 | 1.33 |
| 1996-A | 25.70 | 17.42 | 25.28 | 3.23 | 12.72 | 2.66 | 0.55 | 3.73 | 0.48 | 2.60 | 0.52 | 1.37 | 0.84 | 0.12 |  | 1.28 | 0.81 |
| 1996-B | 46.27 | 67.01 | 120 | 14.13 | 47.76 | 8.38 | 1.52 | 8.04 | 1.07 | 6.00 | 1.18 | 3.16 | 2.20 | 0.31 |  | 1.10 | 0.76 |
| 1997-A | 38.35 | 30.48 | 42.86 | 5.36 | 21.28 | 3.86 | 0.86 | 5.20 | 0.67 | 3.87 | 0.79 | 1.98 | 1.17 | 0.17 |  | 1.27 | 1.09 |
| 1997-B | 29.16 | 26.14 | 36.75 | 4.58 | 18.95 | 3.60 | 0.83 | 4.65 | 0.56 | 3.21 | 0.62 | 1.61 | 1.03 | 0.15 |  | 1.30 | 1.09 |
| 1998-A | 46.24 | 34.98 | 52.55 | 6.47 | 25.46 | 4.99 | 1.18 | 6.37 | 0.81 | 4.65 | 0.94 | 2.41 | 1.27 | 0.20 |  | 1.25 | 1.27 |
| 1998-B | 55.84 | 45.10 | 72.88 | 8.87 | 34.82 | 7.30 | 1.72 | 9.65 | 1.17 | 6.55 | 1.28 | 3.31 | 2.13 | 0.32 |  | 1.31 | 2.26 |
| 1999-A | 50.48 | 41.65 | 63.66 | 7.76 | 29.72 | 5.74 | 1.27 | 6.82 | 0.92 | 5.21 | 1.07 | 2.72 | 1.66 | 0.24 |  | 1.17 | 1.01 |
| 1999-B | 54.47 | 50.34 | 84.12 | 10.76 | 43.20 | 8.67 | 1.87 | 9.60 | 1.23 | 6.85 | 1.33 | 3.53 | 2.46 | 0.36 |  | 1.19 | 1.53 |
| 2000-A | 31.79 | 27.49 | 46.50 | 5.92 | 22.95 | 4.75 | 1.09 | 5.82 | 0.72 | 3.87 | 0.75 | 1.97 | 1.30 | 0.19 |  | 1.26 | 1.20 |
| 2000-B | 32.51 | 25.29 | 42.65 | 5.62 | 22.10 | 4.62 | 1.04 | 5.63 | 0.70 | 3.85 | 0.77 | 2.01 | 1.33 | 0.21 |  | 1.25 | 1.14 |

Supplementary table 2 (continue).

| # | Y | La | Ce | Pr | Nd | Sm | Eu | Gd | Tb | Dy | Ho | Er | Yb | Lu |  | Gd/Gd* | ΔGd |
| --- | --- | --- | --- | --- | --- | --- | --- | --- | --- | --- | --- | --- | --- | --- | --- | --- | --- |
| 2001-A | 44.87 | 40.04 | 59.48 | 7.18 | 28.12 | 5.65 | 1.30 | 7.00 | 0.87 | 4.82 | 0.95 | 2.46 | 1.47 | 0.22 |  | 1.26 | 1.43 |
| 2001-B | 38.56 | 34.15 | 47.18 | 5.74 | 21.44 | 4.20 | 0.93 | 5.48 | 0.65 | 3.62 | 0.72 | 1.88 | 0.96 | 0.15 |  | 1.32 | 1.31 |
| 2002-A | 81.18 | 89.86 | 157 | 20.50 | 81.70 | 16.56 | 3.56 | 16.73 | 2.18 | 11.85 | 2.28 | 5.97 | 4.56 | 0.69 |  | 1.14 | 2.01 |
| 2002-B | 54.23 | 53.55 | 91.81 | 12.15 | 48.43 | 9.87 | 2.11 | 10.74 | 1.34 | 7.42 | 1.41 | 3.66 | 2.65 | 0.40 |  | 1.20 | 1.81 |
| 2003-A | 45.83 | 41.48 | 63.60 | 7.96 | 31.46 | 6.17 | 1.22 | 7.21 | 0.89 | 5.00 | 0.98 | 2.46 | 1.52 | 0.21 |  | 1.24 | 1.40 |
| 2003-B | 47.23 | 39.04 | 61.87 | 8.09 | 31.58 | 6.53 | 1.35 | 7.61 | 0.97 | 5.57 | 1.10 | 2.81 | 1.83 | 0.26 |  | 1.21 | 1.31 |
| 2004-A | 35.26 | 23.51 | 30.81 | 3.93 | 15.20 | 2.81 | 0.65 | 4.64 | 0.51 | 2.86 | 0.59 | 1.47 | 0.56 | 0.09 |  | 1.50 | 1.54 |
| 2005-B | 53.10 | 76.21 | 153 | 18.08 | 66.39 | 12.17 | 2.47 | 10.27 | 1.49 | 8.19 | 1.53 | 3.94 | 3.05 | 0.41 |  | 1.00 | -0.01 |
| 2006-A | 105 | 108 | 189 | 24.92 | 99.58 | 20.37 | 4.05 | 19.29 | 2.75 | 15.46 | 2.99 | 7.90 | 6.59 | 0.98 |  | 1.05 | 0.89 |
| 2006-B | 41.18 | 36.07 | 54.04 | 7.08 | 27.92 | 5.54 | 1.17 | 6.84 | 0.81 | 4.50 | 0.89 | 2.32 | 1.64 | 0.24 |  | 1.29 | 1.56 |
| 2007-A | 33.46 | 41.95 | 79.59 | 9.59 | 35.52 | 6.64 | 1.57 | 5.80 | 0.79 | 4.45 | 0.86 | 2.30 | 1.83 | 0.28 |  | 1.05 | 0.29 |
| 2007-B | 48.87 | 58.46 | 111 | 13.69 | 52.54 | 10.38 | 2.46 | 9.73 | 1.33 | 7.21 | 1.36 | 3.53 | 2.71 | 0.39 |  | 1.08 | 0.69 |
| 2008-A | 45.06 | 36.15 | 54.45 | 6.99 | 28.25 | 5.78 | 1.25 | 7.11 | 0.92 | 5.13 | 1.01 | 2.56 | 1.60 | 0.28 |  | 1.22 | 1.30 |
| 2008-B | 33.61 | 23.47 | 34.63 | 4.31 | 17.36 | 4.25 | 0.80 | 4.96 | 0.60 | 3.53 | 0.70 | 1.83 | 1.24 | 0.20 |  | 1.25 | 0.99 |
| 2009-A | 36.58 | 43.72 | 84.40 | 10.32 | 38.47 | 7.21 | 1.75 | 6.20 | 0.85 | 4.85 | 0.93 | 2.51 | 2.05 | 0.30 |  | 1.04 | 0.23 |
| 2009-B | 33.20 | 52.30 | 105 | 13.12 | 48.99 | 8.87 | 2.03 | 6.75 | 0.88 | 4.76 | 0.89 | 2.33 | 1.91 | 0.28 |  | 1.03 | 0.22 |
| 2010-A | 72.66 | 80.81 | 150 | 18.27 | 68.54 | 13.05 | 3.11 | 11.75 | 1.71 | 9.67 | 1.90 | 5.06 | 4.01 | 0.57 |  | 1.02 | 0.22 |
| 2010-B | 87.58 | 102 | 185 | 22.94 | 84.40 | 15.79 | 3.67 | 14.21 | 1.99 | 11.10 | 2.17 | 5.75 | 4.46 | 0.63 |  | 1.04 | 0.61 |
| 2011-A | 51.55 | 50.99 | 87.97 | 10.54 | 39.12 | 7.24 | 1.74 | 7.09 | 0.99 | 5.67 | 1.14 | 3.07 | 2.31 | 0.33 |  | 1.08 | 0.52 |
| 2011-B | 77.07 | 81.23 | 150 | 17.89 | 66.61 | 12.23 | 2.94 | 10.93 | 1.59 | 9.10 | 1.82 | 4.94 | 3.82 | 0.55 |  | 1.02 | 0.17 |
| 2012-A | 33.49 | 22.47 | 30.53 | 4.01 | 16.37 | 3.18 | 0.68 | 4.87 | 0.54 | 3.36 | 0.64 | 1.69 | 0.97 | 0.15 |  | 1.45 | 1.52 |
| 2012-B | 60.57 | 65.57 | 119 | 14.47 | 53.38 | 9.82 | 2.28 | 8.82 | 1.25 | 7.06 | 1.40 | 3.72 | 2.90 | 0.42 |  | 1.03 | 0.29 |
| 2013-A | 68.65 | 62.78 | 97.25 | 12.18 | 46.81 | 9.04 | 2.15 | 9.61 | 1.32 | 7.38 | 1.45 | 3.73 | 2.58 | 0.36 |  | 1.12 | 1.01 |

Supplementary table 2 (continue)

| # | Y | La | Ce | Pr | Nd | Sm | Eu | Gd | Tb | Dy | Ho | Er | Yb | Lu |  | Gd/Gd* | ΔGd |
| --- | --- | --- | --- | --- | --- | --- | --- | --- | --- | --- | --- | --- | --- | --- | --- | --- | --- |
| 2013-B | 76.14 | 83.78 | 149 | 18.14 | 67.84 | 12.68 | 3.06 | 11.64 | 1.62 | 9.21 | 1.80 | 4.76 | 3.70 | 0.53 |  | 1.05 | 0.59 |
| 2014-A | 60.84 | 64.91 | 113 | 13.81 | 55.67 | 10.23 | 2.32 | 9.98 | 1.35 | 7.70 | 1.43 | 3.73 | 2.71 | 0.38 |  | 1.10 | 0.90 |
| 2014-B | 37.03 | 41.95 | 76.07 | 9.35 | 34.80 | 6.52 | 1.57 | 6.16 | 0.82 | 4.66 | 0.90 | 2.41 | 1.92 | 0.31 |  | 1.09 | 0.53 |
| 2015-A | 20.75 | 21.17 | 40.36 | 5.04 | 18.95 | 3.69 | 0.88 | 3.67 | 0.49 | 2.75 | 0.52 | 1.41 | 1.15 | 0.17 |  | 1.12 | 0.39 |
| 2015-B | 34.16 | 39.57 | 76.69 | 9.41 | 35.03 | 6.62 | 1.65 | 5.98 | 0.81 | 4.51 | 0.86 | 2.31 | 1.92 | 0.28 |  | 1.07 | 0.39 |
| 2017-A | 27.96 | 29.26 | 53.77 | 6.65 | 24.73 | 4.67 | 1.15 | 4.49 | 0.59 | 3.41 | 0.65 | 1.72 | 1.37 | 0.20 |  | 1.11 | 0.44 |
| 2017-B | 51.62 | 30.26 | 43.87 | 5.57 | 22.06 | 4.85 | 1.20 | 7.08 | 1.05 | 6.48 | 1.31 | 3.39 | 2.34 | 0.33 |  | 1.18 | 1.08 |
| 2018-JM1 | 21.38 | 14.51 | 15.92 | 2.13 | 8.87 | 1.89 | 0.44 | 2.51 | 0.34 | 1.92 | 0.36 | 0.95 | 0.80 | 0.10 |  | 1.21 | 0.44 |
| 2018-JM3 | 43.86 | 29.08 | 36.63 | 4.54 | 18.26 | 3.71 | 0.81 | 5.33 | 0.70 | 3.94 | 0.74 | 2.17 | 1.71 | 0.20 |  | 1.28 | 1.16 |
| 2018-SA1 | 11.31 | 6.80 | 7.06 | 1.01 | 4.20 | 0.92 | 0.22 | 1.47 | 0.18 | 0.93 | 0.18 | 0.42 | 0.20 | - |  | 1.39 | 0.41 |
| 2018-A | 40.98 | 36.47 | 45.80 | 5.20 | 19.61 | 3.55 | 0.91 | 4.60 | 0.63 | 3.38 | 0.69 | 1.71 | 0.90 | 0.13 |  | 1.20 | 0.76 |
| 2018-B | 40.21 | 33.86 | 50.25 | 5.84 | 23.50 | 4.64 | 1.12 | 5.76 | 0.75 | 4.13 | 0.80 | 1.98 | 1.24 | 0.18 |  | 1.22 | 1.04 |
| 2018-D | 59.01 | 48.33 | 76.71 | 8.76 | 34.99 | 7.02 | 1.65 | 8.36 | 1.11 | 6.30 | 1.26 | 3.31 | 2.33 | 0.34 |  | 1.19 | 1.33 |
| 2018-E | 37.98 | 29.82 | 41.67 | 5.17 | 20.21 | 4.09 | 0.97 | 5.16 | 0.68 | 3.93 | 0.78 | 2.04 | 1.36 | 0.20 |  | 1.22 | 0.93 |
| 2018-F | 26.12 | 19.08 | 25.94 | 3.20 | 12.59 | 2.62 | 0.63 | 3.58 | 0.44 | 2.68 | 0.51 | 1.35 | 0.90 | 0.16 |  | 1.31 | 0.85 |
| 2018-G | 45.90 | 30.04 | 40.15 | 5.02 | 20.07 | 3.99 | 1.01 | 5.33 | 0.75 | 4.41 | 0.89 | 2.29 | 1.45 | 0.20 |  | 1.19 | 0.84 |
| 2018-I | 51.30 | 40.12 | 57.96 | 7.10 | 28.35 | 5.35 | 1.29 | 6.71 | 0.87 | 5.13 | 1.02 | 2.59 | 1.61 | 0.22 |  | 1.23 | 1.24 |
| 2018-J | 45.77 | 40.11 | 60.44 | 6.96 | 27.93 | 5.35 | 1.24 | 6.53 | 0.84 | 4.95 | 0.98 | 2.54 | 1.69 | 0.26 |  | 1.22 | 1.18 |
| 2018-L | 47.81 | 36.98 | 45.10 | 5.35 | 20.69 | 3.70 | 0.91 | 5.41 | 0.68 | 4.06 | 0.82 | 2.07 | 1.14 | 0.16 |  | 1.31 | 1.29 |
| 2018-M | 45.21 | 36.93 | 46.48 | 5.58 | 21.15 | 3.95 | 0.99 | 5.73 | 0.71 | 4.13 | 0.80 | 1.97 | 1.17 | 0.15 |  | 1.32 | 1.39 |
| 2018-N | 29.51 | 17.85 | 24.78 | 3.31 | 12.89 | 2.72 | 0.66 | 3.85 | 0.47 | 2.88 | 0.57 | 1.56 | 1.02 | 0.16 |  | 1.33 | 0.95 |
| 2018-O | 36.41 | 25.02 | 34.48 | 4.42 | 16.85 | 3.28 | 0.80 | 4.56 | 0.57 | 3.44 | 0.69 | 1.90 | 1.28 | 0.17 |  | 1.30 | 1.06 |

Supplementary table 3. REE+Y abundances (ng/g) in limpet shells from Fuerteventura and Bay of Brest.

| # | Y | La | Ce | Pr | Nd | Sm | Eu | Gd | Tb | Dy | Ho | Er | Yb | Lu |  | Gd/Gd* | ΔGd |
| --- | --- | --- | --- | --- | --- | --- | --- | --- | --- | --- | --- | --- | --- | --- | --- | --- | --- |
|  |  |  |  |  |  |  |  |  |  |  |  |  |  |  |  |  |  |
| *Patella candei*, Fuerteventura, 2017. | | | |  |  |  |  |  |  |  |  |  |  |  |  |  |  |
| PF1 | 11.35 | 11.35 | 7.60 | 1.55 | 6.54 | 1.28 | 0.26 | 1.92 | 0.18 | 1.07 | 0.16 | 0.33 | 0.16 | - |  | 1.60 | 0.72 |
| PF3 | 13.26 | 10.82 | 8.08 | 1.63 | 6.93 | 1.36 | 0.28 | 2.07 | 0.19 | 1.03 | 0.18 | 0.40 | 0.20 | - |  | 1.68 | 0.83 |
| PF4 | 8.98 | 13.68 | 9.13 | 1.80 | 7.29 | 1.31 | 0.32 | 1.81 | 0.15 | 0.80 | 0.12 | 0.25 | 0.14 | - |  | 1.72 | 0.76 |
| PF5 | 15.76 | 12.95 | 9.17 | 2.02 | 8.74 | 1.74 | 0.37 | 2.73 | 0.25 | 1.35 | 0.25 | 0.58 | 0.34 | - |  | 1.66 | 1.09 |
|  |  |  |  |  |  |  |  |  |  |  |  |  |  |  |  |  |  |
|  |  |  |  |  |  |  |  |  |  |  |  |  |  |  |  |  |  |
| *Patella vulgata*, Bay of Brest, 2018. | | | |  |  |  |  |  |  |  |  |  |  |  |  |  |  |
| PM3 | 9.18 | 9.74 | 12.78 | 1.75 | 6.97 | 1.33 | 0.31 | 8.01 | 0.17 | 0.86 | 0.16 | 0.37 | 0.23 |  |  | 6.97 | 6.87 |
| PMOUL3 | 19.01 | 16.82 | 22.06 | 2.86 | 11.23 | 2.31 | 0.53 | 15.14 | 0.37 | 1.96 | 0.37 | 0.93 | 0.65 | 0.08 |  | 6.53 | 12.82 |
| PMOUL4 | 33.39 | 63.63 | 60.29 | 6.44 | 23.48 | 3.57 | 0.77 | 4.99 | 0.53 | 2.59 | 0.47 | 1.01 | 0.46 | 0.04 |  | 1.46 | 1.57 |

**Supplementary references**

(S1) Chauvaud, L. La coquille Saint-Jacques en rade de Brest: Un modèle biologique d’étude des réponses de la faune benthique aux fluctuations de l’environnement, Ph.D. thesis, 265 pp., Univ. Bretagne Occidentale, Brest, France (1998).

(S2) Potts, P.J., Thompson, M., Kane, J.S., Webb, P.C. and Carignan, J. GeoPT6. An international proficiency test. for analytical geochemistry laboratories - report on round 6 (OU3: Nanhoron microgranite) and 6A (CAL-S: CRPG limestone). *Geostandards Newsletter* 24 (1), E1–E37 (200
